# Using Participatory Methods to Create Informational Videos for Inclusive Brain Stimulation Research Recruitment: Action Research Study and Pilot Randomized Controlled Trial

**DOI:** 10.2196/79311

**Published:** 2026-02-09

**Authors:** Marlon L Wong, Lisa M McTeague, Chelsea A Miller, Gabriel Gonzalez, Melissa M Tovin, Frank J Penedo, Eva Widerstrom-Noga

**Affiliations:** 1 Department of Physical Therapy, Miller School of Medicine, University of Miami, 5915 Ponce de Leon Blvd, 5th Floor, Plumer Bldg, Miami, FL, 33146, United States, 1 3052842670; 2 Department of Psychiatry and Behavioral Sciences, Medical University of South Carolina, Charleston, SC, United States; 3 Miller School of Medicine, Department of Medicine, Department of Psychology, University of Miami, Miami, FL, United States; 4 Department of Neurological Surgery, The Miami Project to Cure Paralysis, University of Miami, Miami, FL, United States

**Keywords:** action research, recruitment, consenting, transcutaneous auricular vagus nerves stimulation, transcranial magnetic stimulation, video enhanced recruitment, community engagement

## Abstract

**Background:**

Black and Hispanic/Latino communities experience disproportionate chronic pain and are underrepresented in pain research. Transcutaneous auricular vagus nerve stimulation (taVNS) and transcranial magnetic stimulation (TMS) are promising tools for pain management. Therefore, it is critical to ensure that research using these tools engages all communities to make research findings more generalizable and reach all who may benefit. Lack of diversity in the research workforce itself is a key barrier to improving Black and Hispanic/Latino representation in pain research, and video-enhanced recruitment and consenting may be useful tools to better engage Black and Hispanic/Latino communities.

**Objective:**

The primary goal of this project was to use participatory methods to develop informational videos for inclusive brain stimulation research recruitment.

**Methods:**

Using community participatory research principles in an iterative process, key stakeholders were engaged in 2 consecutive studies to create and then test informational videos on taVNS and TMS. The key stakeholders included neuromodulation researchers as well as Black English-speaking, Hispanic/Latino Spanish-speaking, and Haitian Creole-speaking people with chronic pain. The first study involved iterative feedback from stakeholders through focus groups and interviews to develop test videos, which were then refined based on community member input. The second study was a pilot randomized controlled trial used to assess the impact of these videos on participant expectations for pain relief with taVNS.

**Results:**

Twenty-five community members with chronic pain provided input into the development of the videos, which received overwhelmingly positive feedback. Twenty-eight people with chronic neuropathy were enrolled in the randomized controlled trial, with 24 completing the study. There was no significant difference in expectancy scores between participants who viewed the videos and those who received traditional brochures (median values of 8.2 for both groups; 95% CIs for the means of 7.2‐8.7 and 6.4‐8.7, *P*=.8).

**Conclusions:**

These findings suggest that while the videos may improve engagement, they do not unduly influence expectations, potentially making them valuable tools for improving research participation in noninvasive brain stimulation research. These videos will be freely available to help researchers engage people from diverse communities.

## Introduction

Black and Hispanic/Latino communities are disproportionately affected by chronic pain, with greater prevalence and worse outcomes than non-Hispanic White communities, yet these same communities are underrepresented in pain research [1-9]. Transcutaneous auricular vagus nerve stimulation (taVNS) and transcranial magnetic stimulation (TMS) are forms of noninvasive brain stimulation (NIBS) with great promise for improving pain management. In fact, there is rapidly growing interest in taVNS and TMS, as demonstrated by the >300 clinical trials currently investigating these interventions for pain (listed on ClinicalTrials.gov). However, underrepresentation of Black and Hispanic/Latino communities is particularly problematic in NIBS research due to a number of factors, including lack of diversity in the research workforce, incompatibility between the technologies and certain hair types and styles, and reluctance of Black and Hispanic/Latino populations to participate in clinical trials [10]. This reluctance is often rooted in a well-founded medical mistrust stemming from historical unethical research practices, such as the Tuskegee Syphilis Study, as well as ongoing experiences with systemic bias and a lack of cultural humility within the contemporary health care system [10,11].

It is critical that research on taVNS and TMS includes perspectives of people from these communities to ensure that the research reaches all of those who may benefit and to make research findings more generalizable. Achieving greater diversity in the NIBS research workforce is an important long-term goal for improving this problem. In the present, however, meaningful short-term efforts can be undertaken to improve the inclusion of participants representative of the overall population within NIBS research studies.

A commonly cited barrier to recruiting minoritized people for research is medical mistrust [11]. The best-known approach for overcoming this barrier is community-engaged research [12], and large centers with a history of community-engaged research have been found to offer better opportunities for Black and Hispanic/Latino groups to participate in research [12]. Unfortunately, community-engaged research methods can be challenging to employ, particularly for smaller institutions with limited resources.

Video-enhanced recruitment and consenting can be used to better engage Black and Hispanic/Latino communities, bridging the potential racial discordance between researchers and Black and Hispanic/Latino participants. Recent studies consistently indicate that video enhancement improves participant satisfaction [13,14] and improves understanding and retention of the information provided [15-17]. This is not surprising, as it is widely recognized that many learners experience more effective communication in visual formats such as video. In one of the largest studies on the topic, Fanaroff et al [18] compared the enrollment performance of text-only sites with sites using video enhancement in a multicenter study that included 7904 patients across the United States. Compared with text-only sites, the video enhancement sites enrolled more Black patients. Indeed, a systematic review concluded that “video interventions are well-received by (Black and Hispanic/Latino) survivors and may improve (Black and Hispanic/Latino) representation in clinical trials, yet they are underused.” [13]

Video is increasingly used for getting information in today’s world [19,20]. Information provided in video format is often perceived as more credible [21,22], and, when developed from an inclusive perspective, may help to engender better recruitment and retention rates [13,18]. Such videos may be used for informing communities, recruiting participants, and enhancing the consenting process. However, the development of informational videos must be done with care and with the participation of the communities themselves to avoid missteps that could cause further alienation. Additionally, patient expectations are known to influence pain outcomes [23-26], and it may be important to assess the influence that these videos may have on patient expectations. The primary goals were to (1) involve Black English-speaking, Hispanic/Latino Spanish-speaking, and Haitian Creole-speaking communities to develop informational videos on taVNS and TMS and (2) make these videos publicly available to provide informational materials for the field at large. Collectively, these goals were focused on providing a practical application to establish infrastructure, resources, and community relationships for a research agenda focused on equitable and inclusive research on NIBS for pain management, and the specific research questions (ie, video influence on expectations) were secondary.

## Methods

### Study Design

A “technical-scientific and positivist” [27] model of action research, with the intention of linking research to action, was used for this project. Action research is defined by a focus on generating solutions to practical problems (ie, poor recruitment of Black and Hispanic/Latino persons in NIBS research), and it is characterized by the use of participatory strategies [28]. The technical-scientific and positivist model of action research assumes that the investigators have greater initial research scope than the community participants; thus, in this project, the research questions and theoretical framework were set independently and a priori of interaction with community members, and the community participants acted as “on-the-ground feedback.” [27] By researching and developing culturally sensitive videos, the intention was to provide the research team, and the field, with data and tools to enhance racial and ethnic diversity in NIBS research.

This project consisted of 2 studies. First, an iterative process was used to engage key stakeholders for input and feedback in the video development process (video production study, IRB No. 20230210). Then, the videos were tested in a feasibility pilot randomized controlled trial (Pilot RCT; ClinicalTrials.gov identifier NCT05896202). The pilot RCT was designed specifically to test the videos developed in the first study, and here we focus on the findings pertinent to the videos. Outcomes pertaining to research questions on feasibility, symptom response, and change in physiological measures with taVNS are published elsewhere [29].

### Ethical Considerations

For both studies, approval was granted by the University of Miami Institutional Review Board (20230210 and 20230154), and informed consent was obtained from all participants. Participants in the video production study were compensated US $100 for their participation in the interviews. Participants in the pilot RCT received a compensation of US $250 upon completion of all study activities. We deidentified all data, removed potentially identifying information from transcripts and quotations, and stored files on password-protected systems accessible only to the research team.

### Patient and Public Involvement in the Research

**Figure 1.**
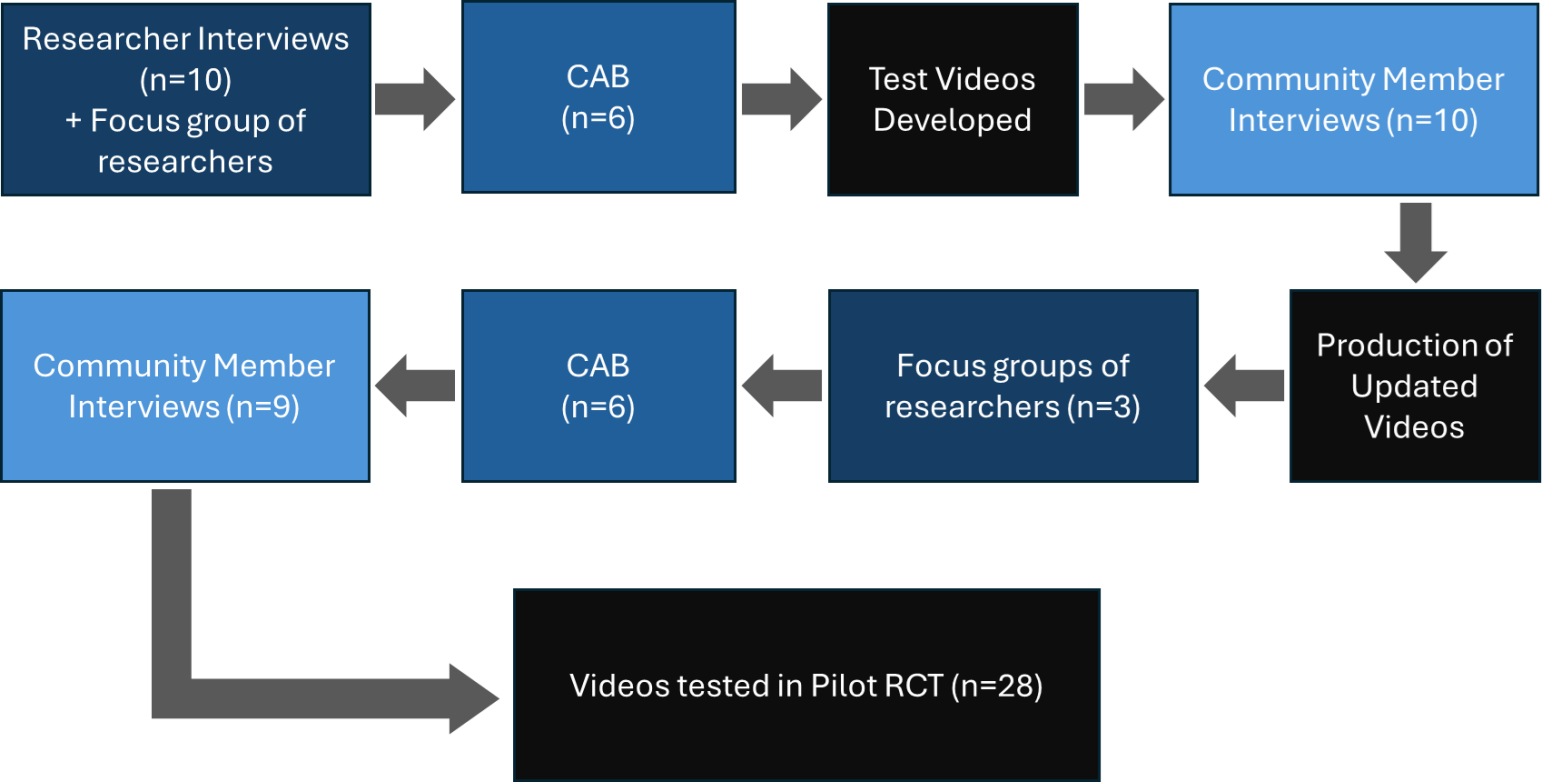
Participant involvement across studies. CAB: community advisory board; RCT; randomized controlled trial.

**Figure 2.**
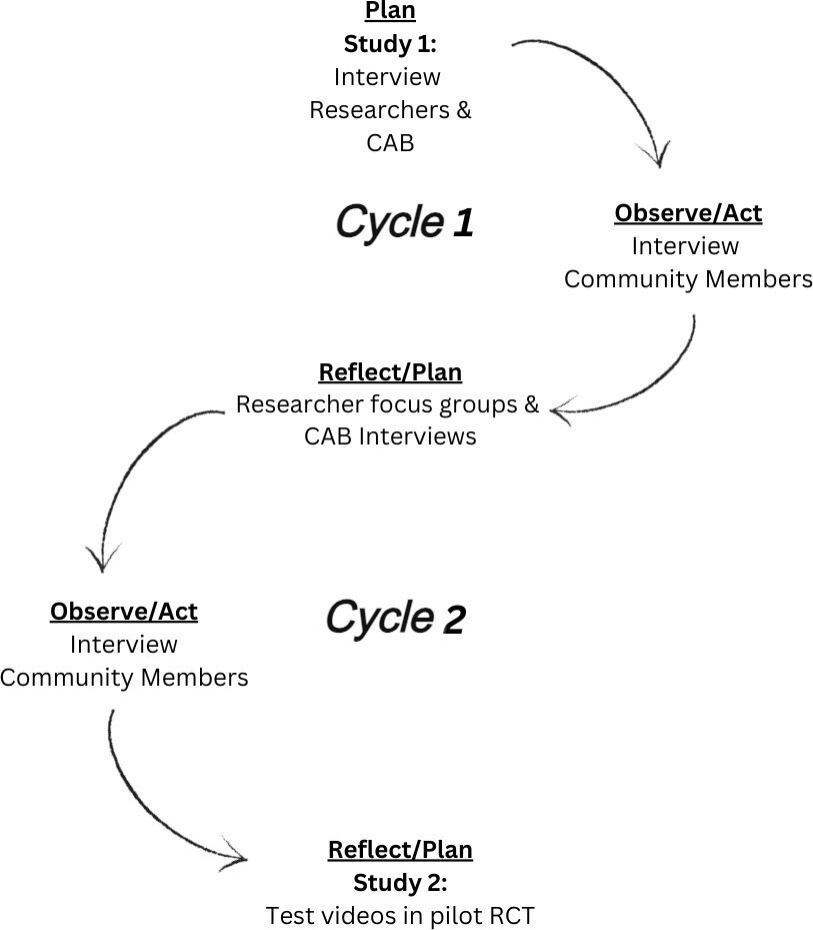
Action research cycle. CAB: community advisory board; RCT: randomized controlled trial.

Two representatives from each ethnic/racial group of interest (ie, Black English-speaking, Hispanic/Latino Spanish-speaking, and Haitian Creole-speaking) were recruited for the community advisory board (CAB), with a total of 6 CAB members. CAB members reported strongly identifying with their respective communities and reported that English, Spanish, or Haitian-Creole was their primary language. CAB members provided feedback on the research plan, recruitment strategy, test and final videos, and the interim summary findings and interpretations. Four of the CAB members also had chronic pain. Additionally, 2 rounds of interviews were used to engage 19 people with chronic pain from the target communities to provide feedback on the videos (Figures 1 and 2).

### Video Production Study

A generic qualitative research design, from an interpretivist paradigm, was employed for this project. This design was chosen because it does not claim allegiance to a single established methodology [30] and thus allowed the researchers to adapt their methods to fit the specific needs of the study as the project unfolded [31]. The interpretivist paradigm emphasizes the subjective nature of reality and acknowledges that individuals create meaning through their experiences and interactions [32,33]. Key stakeholders, including neuromodulation researchers, recruiters, and racial/ethnic Black and Hispanic/Latino community members with chronic pain, were engaged in an iterative process throughout video development (Figures1  2). To get input from neuromodulation practitioners, researchers from the National Center of Neuromodulation for Rehabilitation (NC-NM4R) were first engaged via one-on-one interviews and 2 focus groups. Input from a small focus group of researchers at the Berenson-Allen Center for Noninvasive Brain Stimulation was also obtained during the second round of the feedback process. For Black and Hispanic/Latino community member input, individuals with chronic pain from the representative communities were recruited locally in the Miami-Dade County area through health fairs, flyers in clinics, and snowball sampling to purposefully identify potential participants. Participation was in-person or remote via Zoom, determined by preference.

### Neuromodulation Researchers

As the first step in the project, 60-minute informal, semistructured one-on-one interviews were conducted with 10 researchers from the NC-NM4R, and a large 90-minute focus group of NC-NM4R researchers and recruiters was conducted by the principal investigator (MW), who did not have prior relationships with the interviewees. The interviews and focus group were used to generate ideas for the video content and the desired mood or feel of the video to make sure that the views of those conducting NIBS research were represented in the end product. The format was deliberately informal (ie, no audio recordings) and semistructured to better facilitate idea generation, open feedback, and to encourage diverse perspectives. A sample mood reel was presented to stimulate ideas and feedback, consisting of a 60-second video with sample footage of investigator-participant interactions, animations of neurophysiological effects, and on-screen text. The following questions were used to guide the discussion:

- What are your challenges with recruiting and retaining Black and Hispanic/Latino participants for your research?
- How could videos best be used to help improve the recruitment and retention of Black and Hispanic/Latino participants?
- What content do you think should be in a video [to enhance recruitment and retention of Black and Hispanic/Latino individuals in this type of research]?
- Describe how you envision a short animation would be used to best describe the underlying mechanisms.
- Here is a sample framework for the videos. What are your thoughts?

Using the feedback from researchers, test videos were developed, and these test videos were presented to NC-NM4R researchers via 2 additional informal and unstructured small focus groups for feedback. Feedback was also received from a small focus group of researchers at the Berenson-Allen Center for Noninvasive Brain Stimulation. Field notes were made during and after the interviews and focus groups.

### Community Member Participants

CAB members agreed to meet twice from June 2023 to May 2024. All other community member participants were recruited for a single-session interview and were required to meet the following criteria: (1) be at least 18 years old, (2) have persistent pain of any etiology (eg, back pain, diabetic neuropathy [DN], knee arthritis) for >3 months, and (3) self-identify as one of the target communities and reported that their primary language was English for those in the Black English-speaking group, Spanish for those in the Hispanic/Latino group, or Haitian Creole for those in the Haitian Creole-speaking group. Interviews and focus groups were conducted with racial/ethnic–concordant investigators in the preferred language for each participant. Specifically, all Black English-speaking participants were paired with a Black English-speaking investigator, Spanish-speaking participants were paired with a native Spanish-speaking investigator, and Haitian Creole-speaking participants were paired with a native Haitian Creole-speaking investigator. The interviews and focus groups were audio recorded and transcribed, and non-English recordings were translated into English by a certified professional translation service (GMR Transcription) for analysis. The first round of semistructured interviews was used to gain feedback on the taVNS test videos. Then focus groups of only the CAB members were conducted to provide member checking on interpretation of the interview findings.

In addition to interview or focus group participation, all community member participants completed the questionnaires (in their preferred language) described below.

### Video Production Study Measures

#### Pain Assessment

A structured interview was conducted to determine the participants’ pain characteristics. In addition to questions regarding pain intensity, location, and chronicity, participants were asked to state whether their pain was improving, worsening, not changing, or waxing and waning (pain status).

#### Adapted Group-Based Medical Mistrust Scale (MMS)

Medical mistrust has been widely reported as a key barrier to participation of Black and Hispanic/Latino persons in clinical trials [34]. The Group-Based Medical Mistrust Scale (MMS) was originally developed and has been widely used to assess medical mistrust in Black and Hispanic/Latino people who contact the health care system [35]. The original group-based MMS was adapted to measure mistrust in health researchers (MMS), and the adapted version has also been shown to have strong psychometrics [36]. Thus, the MMS was used in this study to assess community member participants’ degree of mistrust in medical research. The MMS is a 6-item questionnaire assessing a person’s beliefs that their race/ethnic group is prone to mistreatment in medical research. Each item is scored on a 5-point Likert scale ranging from strongly disagree (1) to strongly agree (5). MMS scores are reported as a total of the 6 items, with the last item reverse scored and potential scores ranging from 6 to 30, and higher scores indicate greater mistrust in medical research.

#### Credibility/Expectancy Questionnaire Version II (CEQ*)*

Participants’ feelings of uncertainty pertaining to the credibility and expectancy of researchers and the interventions being studied have also been reported as a barrier to Black and Hispanic/Latino participation in clinical trials. However, the influence of credibility and expectancy on NIBS outcomes has not been established. Thus, the Credibility/Expectancy Questionnaire Version II (CEQ*)* [37] was used to explore credibility and expectancy for pain relief with taVNS. The CEQ is a validated tool for assessing participants’ perceptions on the credibility of therapeutic tools and their expectations for symptom improvement. The questionnaire consists of 6 questions, scored on a Likert scale ranging from 1=Not at all useful to 9=Very useful (with 5 representing “Somewhat useful”). Participants are instructed that the first set of questions pertains to what they *think,* and the second set of questions pertains to what they really and truly *feel*. Examples of the CEQ items include, “At this point, how successful do you think that taVNS will be in reducing your pain?” and “At this point, how successful do you feel that taVNS will be in reducing your pain?” The mean of the 6 items is reported as the CEQ scores, with higher scores indicating greater perceived credibility of taVNS and expectancy for pain relief with taVNS.

#### Protocol for Responding to and Assessing Patients’ Assets, Risks, and Experiences (PRAPARE)

Social determinants of health are known to influence health care utilization and outcomes. The Protocol for Responding to and Assessing Patients’ Assets, Risks, and Experiences (PRAPARE) [38] is a nationally standardized and widely used, screening tool for assessing an individual’s social drivers of health. It consists of 21 items covering the domains of personal characteristics, family and home, money and resources, social and emotional health, and institutional or environmental vulnerability (ie, recent time spent in jail/prison, refugee status, and spousal abuse). In this study, the PRAPARE was used to gather important contextual information on the participants in this study, but it was not scored.

The MMS and PRAPARE were administered at the beginning of each session, and the CEQ was administered after participants viewed the videos and provided feedback.

### Pilot RCT

The pilot study was a single-blinded, sham-controlled feasibility trial designed to (1) examine the influence of the newly developed videos on participant expectations for pain relief with taVNS and (2) explore the feasibility and intended effects of taVNS in Black and Hispanic/Latino people with chemotherapy-induced peripheral neuropathy (CIPN) or DN. A target sample size of 24 was chosen for this study because it was estimated that this would provide the needed power to reach saturation with qualitative analysis and to assess feasibility outcomes [39,40]. Twenty-eight were recruited in all to account for attrition. As noted before, the feasibility and physiological findings are published elsewhere [29], and here the findings pertaining to the influence of videos on participant expectations for pain relief are reported.

The pilot RCT included only Black and Hispanic/Latino patients with CIPN or DN, and block randomization by race/ethnicity was used to ensure that there was equal representation of these groups across the intervention and control groups. Due to financial constraints, we were unable to accommodate the Haitian Creole speakers in the pilot RCT. Participants were recruited from the University of Miami medical health care system from January to May 2024. Potential participants were identified by medical record and then their respective providers (ie, oncologist or endocrinologist) informed them about the pilot study during clinical visits. Inclusion criteria included anyone with glove or stocking distribution paresthesia or dysesthesia that developed after receiving neurotoxic chemotherapies or with a diagnosis of DN and who self-identified as Black or Hispanic/Latino. Exclusion criteria included (1) any unstable medical condition or medical contraindication to moderate physical exertion (eg, unstable angina or cardiac arrhythmia), (2) pregnancy, (3) presence of cognitive impairment or language barrier that impairs full autonomy in the consent process or in the ability to participate in detailed interviews, (4) implants in the head or neck, cochlear implants, or pacemakers, (5) head or neck metastases or recent ear trauma, and (6) history of seizures.

Participants were randomly assigned to video or control groups, and all participants completed 3 visits. Visit 1 consisted of approximately 90 minutes of education on taVNS, including review of brochures and consent forms (both groups) and 3 short video segments on taVNS for the intervention group. The videos contained the same content as the brochures and consent forms, so all participants received the same information but in different formats. Further, all participants had ample opportunity to ask questions and discuss the content with the investigators. Racial and ethnic differences between participant and investigators/providers are also known to influence expectations and pain outcomes [41,42]; thus, a Black investigator provided all educational sessions for Black participants, and a Hispanic/Latino investigator provided all education sessions for Hispanic/Latino participants and in their preferred language (English or Spanish). Both investigators provided the same information to participants. For visits 2 and 3, participants received trials of active or sham taVNS, and those results are described elsewhere.

### Pilot RCT Measures: The Expectations for Complementary and Alternative Medicine Treatments (EXPECT*)*

At the end of the educational session, participants provided feedback on the educational materials and completed the Expectations for Complementary and Alternative Medicine Treatments (EXPECT) [43] questionnaire. The EXPECT is a 4-item questionnaire that assesses expectations for pain improvement. Each of the 4 items is scored on an 11-point scale, with 0 being no change and 10 representing complete relief. Sample items from the EXPECT include “How much change do you hope for in your back pain? and “How much change do you realistically expect in your back pain?’’

### Quantitative Analysis

For both studies, descriptive statistics were assessed for demographic data and questionnaire findings. This included sample means, medians, and SDs for each continuous variable and frequencies and percentages for categorical variables. Group comparisons were conducted using Kruskal-Wallis and chi-square tests, and correlations were assessed using Spearman ρ. Statistical analyses were conducted using SPSS (version 29; IBM Corp.), and figures were rendered using GraphPad Prism (version 10.4.0; Graphpad Software).

### Qualitative Analysis

Rapid qualitative analysis is widely used for implementation projects, such as this, when the goal is to create change in response to the findings rather than to generate new theories. Rapid qualitative analysis was systematically applied in this study according to established protocols [44]. The interview guide was used to create structured templates and matrix displays to facilitate data condensation, synthesis, and theme development. Templates were developed collaboratively by the team (MW, CM, CG) and pilot tested to ensure consistency, usability, and relevance. Once consistency was demonstrated, MW completed summaries of transcripts from sessions with Black English-speaking participants, both GG and CM completed summaries of transcripts for Spanish-speaking participants, and CM completed summaries of transcripts for Haitian Creole-speaking participants. The summaries were aggregated by MW and CM to populate the matrices that enabled systematic comparison between participants.

All 3 team members who were engaged in the qualitative analysis were physical therapists and pain scientists, with an interest in health equity and with extensive experience working with the target populations in clinical settings. As mentioned earlier, all recordings were transcribed in English, and the racial and ethnic backgrounds of the team members who analyzed the transcripts were Black Caribbean (MW, male), White American (CM, female), and Latino Mexican (GG, male). MW had experience with qualitative and mixed-methods research through involvement in several funded projects, and he received formal mentorship in qualitative research from recognized experts as part of the pilot funding programs that supported this project. CM had completed formal coursework and training in qualitative research as part of attaining her master’s degree in public health. This was GG’s first exposure to qualitative research, and he received training in interviewing and qualitative research principles prior to initiating data collection processes.

## Results

### A. Development of Test Videos

#### A.1 Neuromodulation Researchers’ Initial Input for Video Content

A variety of perspectives were gained from the informal interviews and focus group with NC-NM4R researchers. Most felt that the best use of the videos would be for recruitment purposes, while some felt that they would best help with enhancing the consent process, and a few felt that the best use of videos would be to direct them to the researchers, enhancing their cultural sensitivity. Recruitment was commonly viewed as the biggest barrier. Thus, the decision was made to design the videos to be primarily informational, to assist with recruitment efforts.

Opinions also differed on the optimal length and content for the videos, with some advocating for very brief videos (<1 min) to capture attention and generate interest with recruits, and others advocating for more lengthy videos (over 5 min) to provide detailed information on the research procedures as well as the risks and regulations involved. As a result, we decided to start with the taVNS content and create test versions for full-length videos (~6 min in length) as well as segmented versions, in which the full-length videos were divided into 3 separate video segments organized around the following content areas: (1) introduction and how taVNS works, (2) risks and regulations, and (3) what to expect when you participate in taVNS research (Table 1). In the introduction segment, information on the potential indications for taVNS was provided, and an animation of the proposed mechanisms of taVNS was presented. In the risks and regulations segment, the potential side effects were outlined, and the process of research oversight was described. In the final segment, entitled *what to expect*, the process of prepping and applying taVNS was demonstrated. Block allocation was used, with alternating assignment within each racial/ethnic group (ie, the first participant received the full-length version, and the second participant received the segmented version for the Black English-speaking group), to get feedback on the full length and segmented versions of the videos.

**Table 1.** Overview of test video segments.

Video segment	Content summary	Key imagery
1. Introduction and how it works	Potential effects include decreased pain, improved mood, and enhanced brain function. Based on technology that has been safely used for over 60 years. Description of the vagus nerve anatomy and physiological effects.	[ 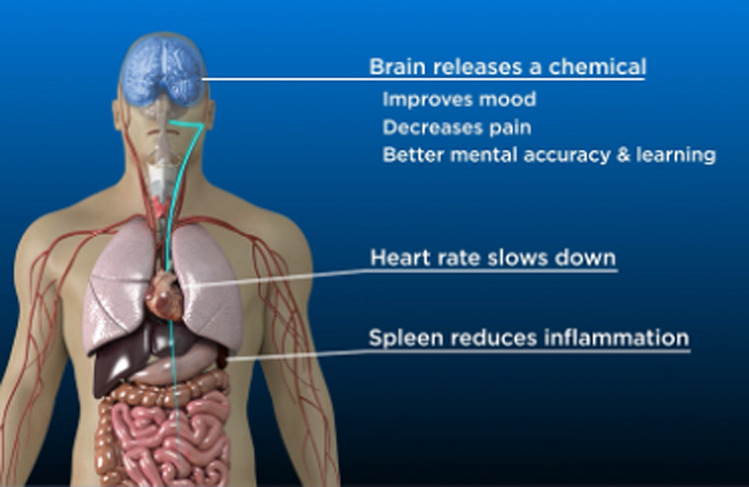
2. Risks and regulations	Additional research is needed to gain FDA^a^ approval for the noninvasive form of taVNS^b^. Studies are done with strict oversight to ensure that they are being done safely and ethically. There are no guarantees that it will help you. It has been shown to be very safe thus far with low rates of side effects.	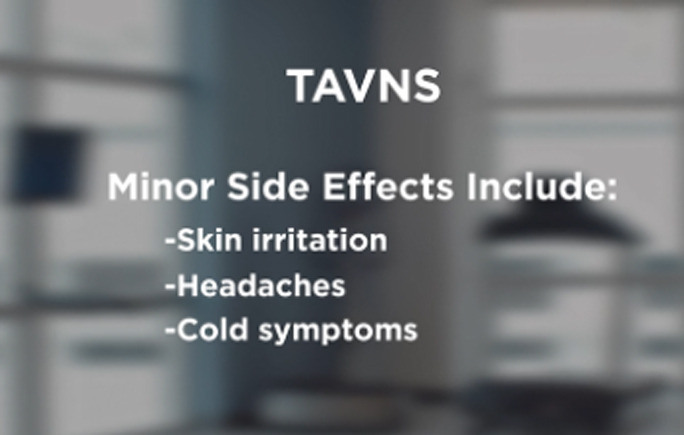
3. What to expect	You will set up in a comfortable position, and they will clean around the ear to apply electrodes. In some studies, the stimulation is so mild that you don’t feel anything at all, and in other studies, the stimulation may be set to an intensity that you feel as a strong tingling sensation. Heart rate monitoring is often used with taVNS to assess the effects.	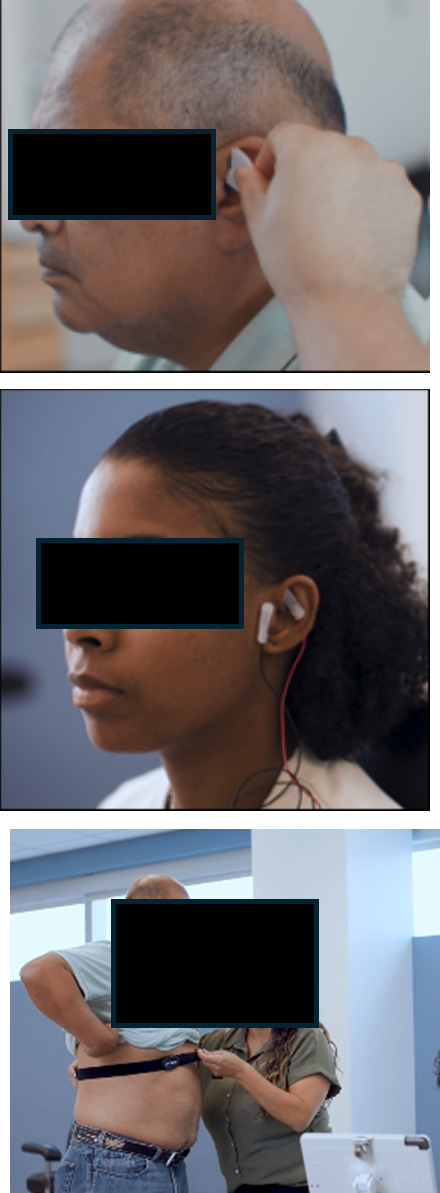

a FDA: Food and Drug Administration.

b taVNS: transcutaneous auricular vagus nerve stimulation.

#### A.2 Community Member Participants: Round 1

Sixteen community members, including both CAB and community participants with chronic pain, provided feedback on the test videos produced in their respective languages (5 Black English-speaking, 6 Spanish-speaking, and 5 Haitian Creole-speaking). The group mean (SD) age was 51.6 (16.2) years, and 12 of the participants were female (12/16, 75%). The 14 participants with chronic pain had moderate pain on average, with mean (SD) scores on a scale of 0 to 10 for current, best, and worst pain intensity within the last week of 4.9 (3.2), 3 (2.7), and 6.9 (3.4), respectively. There were no significant differences between racial and ethnic groups in other demographic characteristics, pain characteristics, or social risk factors (Table 2). The group was diverse in social risk factors, with 3 of the participants not having any health insurance, 3 with Medicaid coverage, 5 with Medicare, and 5 with private insurance. Four participants reported that within the last year, they or a family member they live with were unable to get medicine when it was needed. There was also diversity in medical mistrust, with a group mean (SD) score on the MMS of 17.8 (6.6), a median of 18.5, and scores ranging from 6 to 26.

**Table 2.** Participant characteristics.

Primary language	Sex	Age (y)	Diagnosis	Current pain	Best pain	Worst pain	Pain status
Round 1							
English	Female	76	Knee pain	6	2	8	Not changing
English	Female	66	Lumbar degenerative joint disease	3	3	10	Getting worse
English	Male	52	Postherpetic neuralgia	2	1	6	Getting better
English	Female	59	Spinal Stenosis	8	9	2	Getting worse
English	Female	68	Arthritis	0	0	7	Coming and going
English	Female	79	Arthritis	7	3	10	Not changing
Spanish	Male	36	—^a^	—	—	—	—
Spanish	Female	46	Lumbar herniated disc	10	8	10	Getting worse
Spanish	Female	31	Migraine	9	2	8	Getting worse
Spanish	Female	38	Scoliosis	6	3	10	Coming and going
Spanish	Male	42	Back pain	7	3	10	Coming and going
Haitian Creole	Female	29	—	—	—	—	—
Haitian Creole	Female	59	Lumbar radiculopathy	4	2	7	Coming and going
Haitian Creole	Female	58	Abdominal pain	4	1	8	Not changing
Haitian Creole	Male	30	Angina	6	5	8	Coming and going
Haitian Creole	Female	56	Arthritis	6	4	6	Coming and going
Round 2							
Spanish	Female	62	Lateral epicondylitis	4	3	9	Coming and going
English	Female	72	Osteoarthritis, Congenital spinal stenosis	10	1	10	Getting worse
Spanish	Female	46	Low back pain	5	3	8	Not changing
Haitian Creole	Female	52	Foot pain	4	—	—	Not changing
English	Female	37	Plantar fasciitis	5	3	10	Getting worse
Haitian Creole	Male	74	Headaches	0	0	0	Coming and going
Haitian Creole	Female	65	Knee pain	5	3	8	Getting worse
English	Female	37	Ankylosing spondylitis	7	5	10	Coming and going
Spanish	Female	49	Shoulder pain	6	5	8	Not changing

a Not available.

The interviews identified several potentially important differences across racial and ethnic groups for participants’ current pain management strategy. All Black English-speaking participants used medication as their primary pain management strategy, and none had ever tried electrical stimulation. Conversely, 4 of the 5 (80%) Spanish-speaking participants used medication for pain management when needed, 2 of the 5 (40%) had tried electrical stimulation, and all reported cognitive/emotional or active coping strategies (ie, distraction, “learning to live with it,” breathing, going to the gym) as primary forms of pain management. Three of the 5 (60%) Haitian Creole speakers reported using medication for pain when needed, and all reported using alternative or “natural” treatments (ie, tea, massage, oil) as their primary pain management approach.

#### A.3 Community Member Participant Feedback on Test Videos

Participants watched the videos in their primary language and provided feedback in their primary language (ie, American English, Caribbean Spanish [since these videos were intended to be used in Miami-Dade County], and Haitian Creole). Overall, the videos were well received by participants, and details on their feedback and the decision log can be found in Supplement 1 in Multimedia Appendix 1. Key themes identified across all groups were that they most appreciated the animation and education on “how it works,” they found the video to be “clear,” and there was concern about taVNS not being Food and Drug Administration (FDA) approved. The general appreciation for the videos’ educational value is best articulated by the following participant comment:

*This is a positive all the way across the board, because I can tell you it by being a black female and dealing with different doctors. Most of them tell you, they don't talk to you. They don't allow time for you to ask questions, or just like you had a video to show.*

[P-E3]

In addition to frequent and repeated use of the term “clear,” the feeling that the video was comprehensible was supported by comments such as “good pace,” “educational,” “enough info and easy to understand.” The concern about taVNS not being FDA approved was commonly expressed by participants across racial/ethnic groups.

The mean (SD) participant CEQ score was 7.2 (1.2) (median 7.2), with scores ranging from 5 to 9, indicating that all participants rated the credibility and expectancy as moderate to high. There were no differences between racial and ethnic groups in CEQ scores (*P*=.57), and CEQ scores were not significantly correlated with MMS scores (*r*=.06, *P*=.83). Participant feedback was similar for both the full-length and segmented versions of the videos. One participant thought that the full-length version was too long to digest in a single viewing; additionally, the segmented versions seemed to encourage more participant interaction with the investigators between segments, and this enhanced engagement may facilitate rapport building and recruitment efforts.

### B. Development of Final taVNS and TMS Videos

#### B.1 taVNS Video Edits in Response to Feedback

The original plan was to reshoot the videos in response to participant feedback. However, feedback was overwhelmingly positive, and thus the videos received only minor editing alterations. For example, due to the enhanced engagement of the shorter videos, we decided to only make segmented versions for the final taVNS and TMS video production. In addition, to allay participants’ concerns about FDA approval, we added the following to the narration of the taVNS Risks and Regulations video segment:

*An invasive form of vagus nerve stimulation is FDA approved to treat epilepsy, depression, and stroke. Additional medical research is needed to gain FDA approval for the noninvasive form of TAVNS with these conditions and for other conditions like chronic pain*.

#### B.2. TMS Video Development

Financial constraints were a major limiting factor, and we were only able to develop videos on TMS in English and Spanish due to the high costs associated with video production, conducting interviews and focus groups on Haitian Creole, and for translation and transcription services. We applied lessons that were learned from the test video development for taVNS (ie, preference for segmented versions and concerns about FDA approval), and the TMS content was divided into 4 short video segments entitled (1) introduction (how TMS works), (2) TMS for research, (3) TMS as a treatment, and (4) risks and regulations.

The final videos are available for public use, and the web addresses for the taVNS and TMS videos can be found in Multimedia Appendix 2.

#### B.3. Final Video Feedback: Round 2

To get feedback on the final videos, 3 new community members from each racial and ethnic group were interviewed (Table 2). Since there was no additional budget for translation services, feedback was sought from bilingual Spanish and Haitian Creole speakers for the final round of interviews, and they watched the videos in their primary language but provided feedback in English. The bilingual Haitian Creole speakers provided feedback on the final taVNS videos in Haitian Creole and TMS video in English since Haitian Creole versions of the TMS videos were not produced. Participants in the second round of interviews had lower levels of mistrust than those in the first round, with mean (SD) MMS scores of 11.2 (3.2) (median 12, range 6‐15)

Overall, the videos for both taVNS and TMS videos were well received, and details of their feedback and preferences can be found in Supplement 1 in Multimedia Appendix 1. For the taVNS videos, the mean (SD) participant CEQ score was 7.7 (1.1) (median 7.9), with scores ranging from 5.7 to 9, indicating that again all participants rated the credibility and expectancy as moderate to high. Once again, participants appreciated the clarity of the videos, calling them “clear,” “concise,” and “transparent.” In addition, word choice and translation were important to the Spanish-speaking group. Specifically, they commented that although the translations used for the words “safety” and “seizure” were accurate, the literal translation was more alarming in Spanish.

After providing feedback on the videos for both taVNS and TMS, participants were asked if they were more interested in trying one of these interventions than the other. Key themes regarding treatment and learning preferences across groups were that they preferred the treatment option of TaVNS compared to TMS. All participants expressed greater interest in taVNS, and many indicated that there was a lack of clarity for how TMS would help with pain. For example, participants stated:

*The first one you literally stated it could be effective with the chronic pain conditions. Especially, for me, the inflammation. Whereas the second one, it was more so for the smoking or the obsessive-compulsive disorder*.

[P-E7]

*…Because the TMS helps with convulsions, depression, and things like that I didn’t feel it’s something that can help me with my chronic joint issue, with what I have*.

[P-S8]

This is also supported by the greater detail provided by participants in their descriptions of what they learned from the taVNS videos compared to the TMS videos. Many participants were able to discuss specific details on the theorized mechanisms for pain reduction via the vagus nerve with taVNS, but they could only vaguely discuss connections between brain stimulation and pain reduction with TMS.

Brochures of the same video content were also produced, and participants were asked to provide feedback on their preference for learning about taVNS and TMS from the videos or brochures. Surprisingly, there were varied preferences. All 3 Spanish-speaking participants preferred learning from the video because you can see the procedure and participants undergoing the procedure, stating “you see more of what will happen to you” [P-S6], and “you see that the patient is calm…” [P-S7]. However, 2 of the 3 Haitian Creole-speaking participants preferred the brochures over the videos. When providing reasons for their preference of the brochure over the video, the 2 participants discussed the importance of being able to take their time to read and research the information to discuss with their physician to make a decision about their care. For example:

*I like the reading part. The video will be limited to me because even if it’s on TV or if you watch the video. But if I got it on my hand, I can read it, I can read it again, and then I can get that with me, and sit down somewhere, do more research…I keep the paper on my hand and I write it down. I notice word, which is something about what I don’t understand, I can go verify what’s the meaning of that…*

[P-C4]

*You grab one, you read it, that’s how you are going to know the medication, that’s how when I go to the doctor’s I tell my doctor that I read the brochure outside and I see this medication is good for this certain thing*.

[P-C3]

### C. Testing of the taVNS Videos in a Pilot RCT

**Figure 3.**
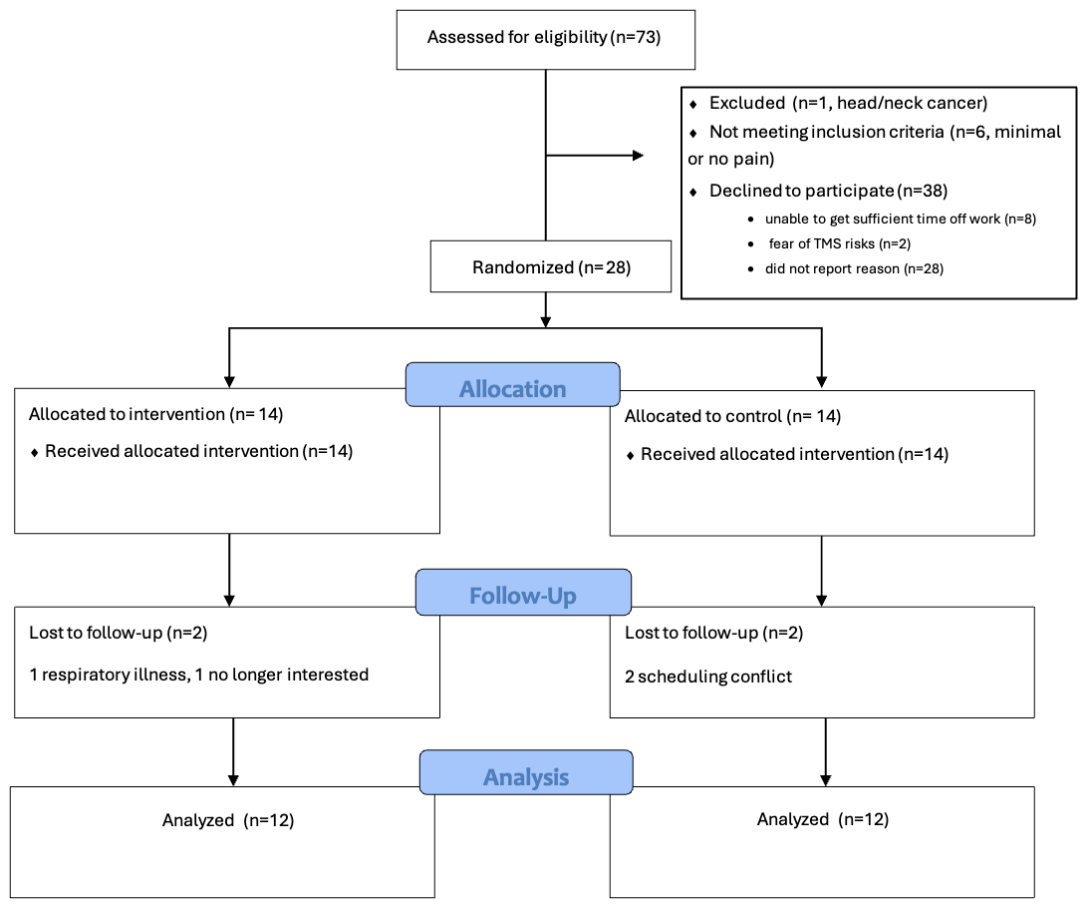
CONSORT (Consolidated Standards of Reporting Trials) diagram. TMS: transcranial magnetic stimulation.

Twenty-eight people with peripheral neuropathy were recruited (Figure 3; Checklist 1), of whom 14 received the taVNS videos in their preferred language (video group), and 14 received education via brochures only (control group). There were no differences between groups in participant demographics (ie, race/ethnicity, sex) or medical condition (CIPN vs DN). The mean (SD) age of the participants was 58.2 (11.3) years; 20/28 (71%) were female, and 14/28 (50%) identified as Black and 14/28 (50%) identified as Hispanic/Latino. Additionally, 17/28 (61%) had CIPN, and 19/28 (68%) were using prescription medication to manage neuropathic symptoms, with gabapentin the most used medication (17/28, 61%). On average, participants had high symptom burden with mean scores ranging from 6.1 to 8 on a scale of 0 to 10 for each of the following symptoms: pain, numbness, tingling, burning, and shooting or electric shocks.

**Figure 4.**
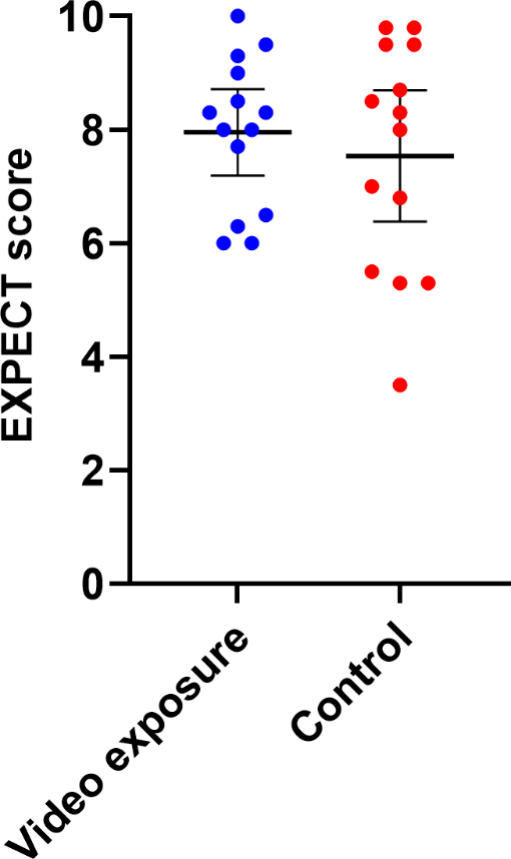
Group differences in EXPECT scores. EXPECT: Expectations for Complementary and Alternative Medicine Treatments; taVNS: transcutaneous auricular vagus nerve stimulation.

After receiving education on taVNS, there was no meaningful difference in the median EXPECT scores between the video and control groups, with respective mean values of 8 and 7.5 (median values of 8.2 for both groups, and 95% CIs for the means of 7.2‐8.7 and 6.4‐8.7, *P*=.8). However, the video-exposed group had less variability in EXPECT scores, with an SD of 1.3 compared with 2 for the control group (Figure 4). Similarly, there were no differences between groups after completing the trial, with mean (SD) EXPECT scores at visit 3 of 8.1 (0.9) and 8.2 (1.9) for the video and control groups (median values of 8.1 and 9 and 95% CIs for the means of 7.6‐8.7 and 6.9‐9.4; *P*=.6). Further, there was no significant change between visit 1 and visit 3 EXPECT scores (mean difference −0.2, *P*=.28).

## Discussion

### Principal Findings

In these 2 studies, community participatory research principles were used to develop and test culturally sensitive informational videos on taVNS and TMS. These videos were designed specifically to enhance the recruiting and consenting processes for taVNS and TMS research with Black English-speaking, Hispanic/Latino Spanish-speaking, and Haitian Creole-speaking people by providing the information in an easily digestible format, with input from the target communities, and with representation of these communities within the videos. The final video products were well received and generated interest in these modalities among the participants. We anticipated that the videos would increase expectancy for pain relief with taVNS, but there were no differences in EXPECT scores between those who viewed the videos and those who learned about taVNS from brochures only. Although unanticipated, this finding likely increases the potential value of these videos for use in research. An undue increase in expectations could be a potential confounding factor in studies, and these results suggest that these videos are neutral and do not generate unrealistic or misleading expectations for pain relief with taVNS.

A strength of this study was the range of medical mistrust observed in the sample, with scores from the lowest possible to the highest possible on the MMS. In a racially and ethnically diverse sample of 615 American adults and adolescents, the mean MMS score was 13.3 [36], which is lower than the mean score of 15.8 found in this sample. Much has been made of medical mistrust as a key barrier to recruitment and retention of Black and Hispanic/Latino groups in research; however, in this study, high medical mistrust did not result in low perceived credibility, expectancy, or interest in participating in taVNS research. Feelings of uncertainty are known to contribute to Black and Hispanic/Latino patients’ reluctance to participate in research, and poor-quality information can contribute to participants’ uncertainty [45]. Research on Media Richness Theory has shown that richer media are viewed as more credible, and that a video-with-audio medium will be perceived as higher in credibility than a picture-with-text medium [21,22]. Therefore, it is plausible that the high CEQ scores and expressed interest in taVNS research observed in this study were a result of the videos mitigating feelings of uncertainty. However, additional research is needed to confirm this.

It was interesting that all community participants in round 2 of video production study expressed greater interest in taVNS than TMS, and they demonstrated greater understanding of the proposed mechanisms for pain relief with taVNS compared to TMS. Therefore, in this sample of chronic pain patients, understanding treatment mechanisms appeared to be an important factor in treatment preference across cultural/language groups. There were also group differences in their preference for learning material format, with only members of the Haitian Creole-speaking group expressing a preference for brochures over video. It is well known that Haitian immigrants have complex language and cultural barriers that limit access to health care services [46-48]. Haitian participants in this study differed from the Black English-speaking and Hispanic/Latino Spanish-speaking participants in that they preferred brochures over videos for learning about NIBS, and this highlights the importance of using nuanced approaches to optimize community engagement in research. We plan to build on these lessons in the future by providing future potential Creole-speaking participants with brochures in both Haitian Creole and in English, so that they will have tools to discuss the studies with their families and providers.

### Limitations

Although these studies achieved their aims, there are key limitations worth noting. The qualitative analyses were done on translated transcripts of the Spanish and Haitian Creole audio recordings rather than transcripts in the native languages. Additionally, no Haitian Creole speakers were involved with analyzing the transcripts. It is possible that salient points were missed or misinterpreted during translation, but this risk was mitigated by member checking during CAB meetings. It is also important to note that while many of the participants were originally from different countries or different parts of the United States, all but one were current residents of the Miami-Dade County area. Thus, the findings in this study may not be reflective of all other communities, and researchers should consider the nuances of their respective communities and possibly test the videos before implementing them.

Due to budgetary constraints and the high costs associated with translation services, we were unable to produce Haitian Creole versions of the TMS videos. Outside of costs associated with video production and personnel for conducting the interviews, over US $17,000 was spent on translation and transcription services across both studies, with more funds dedicated to Haitian Creole translation than either English or Spanish, despite not including Haitian Creole speakers in the pilot RCT. For example, the rates that were received for transcribing the audio recordings of the interviews varied greatly depending on the language, with the per recorded minute rate at US $1.50 for English-to-English, US $5.50 for Spanish-to-English, and US $20 per audio minute for Haitian Creole-to-English. This 13-fold increase in transcription costs for Haitian Creole audio is a significant barrier to including this underserved population in health research opportunities. Further, compared to the English and Spanish recordings, the transcription service used was more likely to deem the audio quality of the Haitian Creole interviews as “difficult,” which added US $0.50 per minute to the cost. Based on a review of transcription service websites, language is one of the greatest factors in determining cost, with less common languages costing more. Artificial intelligence (AI) tools have the potential to transform this disparity by providing access to cost-effective transcription. However, currently, human transcription is more accurate than AI, and existing AI services do not cover less common languages like Haitian Creole [49].

Our goal was to use participatory research principles in this research, and we implemented this by using the CAB and seeking input from community members. Ideally, we would have used a more mutually collaborative model, in which community members have input throughout the research process, including conceptualization, data collection, and dissemination of the findings. However, these studies were the first steps in developing infrastructure for a research agenda focused on the use of NIBS for equitable pain management; thus, we did not have strong existing relationships with community partners, and most of our participants were not experienced with research involvement. Additionally, community participants expressed difficulty generating unguided input due to the novelty of taVNS. As a result, we chose to use a technical-scientific and positivist [27] in which community members were only used as a sounding board for feedback.

### Recommendations for Future Research and Programming

Based on the findings of this action research study, the following recommendations are proposed for future research and recruitment initiatives.

#### Prioritize Mechanism-Based Education

Recruitment materials should clearly explain the neurophysiological mechanisms of the intervention (eg, how taVNS affects the vagus nerve), as participants reported that understanding “how it works” significantly enhanced treatment credibility and interest.

#### Implement Nuanced Cultural Tailoring

Researchers should avoid a monolithic approach to minority recruitment. For instance, while video was preferred by Black English-speaking and Hispanic/Latin Spanish-speaking participants, Haitian Creole speakers in this study preferred traditional brochures, highlighting the need for community-specific formats.

#### Mitigate Uncertainty Through Rich Media

Using high-quality video content can help mitigate feelings of uncertainty and medical mistrust by providing transparent, accessible information that is perceived as more credible than text-only materials.

#### Transition Toward Collaborative Models

Future programming should move from “technical-scientific” feedback models toward deeply collaborative partnerships that involve community members in the early conceptualization and design of research protocol.

### Conclusions

Together, these 2 studies describe the process of engaging stakeholders to develop and test culturally sensitive informational videos on taVNS and TMS. The iterative process used to develop the videos in this project resulted in enhanced community awareness, engagement, and interest in our research agenda. Our hope is that the videos produced in this project will provide NIBS researchers with culturally sensitive and useful tools to engage Black and Hispanic/Latino communities in their research.

## Supplementary material

10.2196/79311 Multimedia Appendix 1 Qualitative data matrices.

10.2196/79311 Multimedia Appendix 2 Video links.

10.2196/79311 Checklist 1 CONSORT checklist.

## Abbreviations

AI: artificial intelligence
CAB: community advisory board
CEQ: credibility/expectancy questionnaire
CIPN: chemotherapy-induced peripheral neuropathy
DN: diabetic neuropathy
EXPECT: Expectations for Complementary and Alternative Medicine Treatments
FDA: Food and Drug Administration
MMS: Medical Mistrust Scale
NC-NM4R: National Center of Neuromodulation for Rehabilitation
NIBS: noninvasive brain stimulation
PRAPARE: Protocol for Responding to and Assessing Patients’ Assets, Risks, and Experiences
RCT: randomized controlled trial
taVNS: transcutaneous auricular vagus nerve stimulation
TMS: transcranial magnetic stimulation
