# Supplementary material for: Using Participatory Methods to Create Informational Videos for Inclusive Brain Stimulation Research Recruitment: Action Research Study and Pilot Randomized Controlled Trial
Source: J Particip Med. 2026 Feb 9;18:e79311. doi: 10.2196/79311 (PMC12885457; doi:10.2196/79311)
Supplement: Multimedia Appendix 1 [file jopm-v18-e79311-s001.docx]

| Multimedia Appendix 1 | | | | | | | | | | | | | | | | | | |  |
| --- | --- | --- | --- | --- | --- | --- | --- | --- | --- | --- | --- | --- | --- | --- | --- | --- | --- | --- | --- |
| English  Round 1 Interviews: Video Feedback | Participant | | | What Was Learned from the Video | | | | Positive Video Feedback | | | | | | Negative Video Feedback or Additional Information | | | | |  |
|  | CAB1 | | | “besides the pain, I also had high blood pressure as well.” | | | | - Good pace - Honest and direct - segmented | | | | | | - Why did people drop out? - Say that it has been FDA approved for other things - Say the contact info at end | | | | |  |
|  | CAB2 | | | - Not FDA approved - Improvement on TENS - How it works (animation) | | | | - Enough info, easy to understand for younger people - To the point and not too long - Good narration | | | | | | - Concern about not being FDA approved - Concern about putting electricity in the body | | | | |  |
|  | P-E1 | | | - That it's one of the main nerves that goes down to the core of the body and regulates things like heartbeat. | | | | - Animation was excellent - Everything's professional. It's well done. Transparent. | | | | | | “say, well, this is not like that. It's not going to be harsh shock. You're not going to be electrocuted.” | | | | |  |
|  | P-E2 | | | - Techniques, what it affects, sensations - Good for BP and heart rhythm | | | | - Very good study - Interested in it for herself because of arteriosclerosis/BP | | | | | | - Add testimonials of people who experienced it | | | | |  |
|  | P-E3 | | | - Noninvasive - How it works (animation) | | | | “positive all the way across the board…most [MDs] don’t allow time for questions or have a video to show” | | | | | | - Diversify (older women, Asian). Ageism. | | | | |  |
|  | P-E4 | | | - Vagus nerve - What it entails - Something I could try for my high BP, anxiety, digestion | | | | - Pretty clear to me | | | | | | - Go into more depth: what’s damaged | | | | |  |
|  | *Key Themes Across Participants* | | | - *Focus on the "how it works" and animation.* - *Important aspects include: noninvasive, cardiovascular effects* - *Slow pace is important for clarity* | | | | | | | | | | | | | | |  |
|  | | | | *Decision Log for Video Enhancement (Based on Feedback):* | | | | | | | | | | | | | | |  |
|  |  |  |  | No Action | | | | | | | - Why did people drop out? - Diversify - Go into more depth on what's damaged - Add testimonial of people who experienced it - Concern about putting electricity in the body | | | | | | | |  |
|  | | | | Action | | | | | | | - Concern about not being FDA approved (uncertainty) | | | | | | | |  |
| English  Round 2 Interviews: Feedback and Expectations |  | | TaVNS | | | | | | | TMS | | | | | | | Preference |  |  |
|  | Participant | | What Was Learned from the Video | | | Video Feedback | Brochure Feedback | | | What Was Learned from the Video | | | Video Feedback | | | Brochure Feedback |  |  |  |
|  | P-E5 | | - Knew about TENS but learned about the vagus nerve and electrical stimulation. | | | Positive:   - Clear, concise - Side-effects were clear   Areas for Improvement:   - None | Positive:   - Good color - Easy to read - Covers what the video covers.   Areas for Improvement:   - Consistency with TaVNS/TAVNS - Different image than video, use one for consistency. | | | - It has been used for 15 years to help in other areas. | | | Positive:   - Clear   Areas for Improvement:   - Include length of time for treatment - Clarify ear damage with earplugs or without. | | | Positive:   - Clear   Areas for Improvement:   - All one color - Incorporate earplug statement. | TaVNS vs. TMS:   - TaVNS at home. |  |  |
|  | P-E6 | | - Vagus nerves impact on spleen and inflammation - Regulate pain through HR and relaxed state - Mild side effects - Learned the most from diagram | | | Positive:   - Short, clear, to the point. - Enjoyed the transparency.   Areas for Improvement:   - None stated. | Positive:   - Good - Pictures are helpful   Areas for Improvement:   - None stated | | | - Learned that it is working to restore balance in the brain. | | | Positive:   - Descriptive - Seizure risks explained well - Connecting why the finger twitching correlates with everything. - Has had TMS before, feels this video was most inviting and informative than previous experience.   Areas for Improvement:   - How it is specifically related to pain. | | | Positive:   - Good - Diagram use and real person were good   Areas for Improvement:   - None | TaVNS vs. TMS:   - TaVNS because of the spleen and inflammatory connection. |  |  |
|  | P-E7 | | - Vagus nerve is the longest nerve. - Vagus nerve can control inflammation. - Learned the most from the first video. | | | Positive:   - Clear   Areas for Improvement:   - Directly state diverse group of people are needed for accurate data. - Any comorbidities restrict from participating. | Positive:   - Pictures and information are good.   Areas for Improvement:   - Brighter colors | | | - Feels the TaVNS video explained more and did not describe what was learned from TMS. | | | Positive:   - Videos clear.   Areas for Improvement:   - Incorporate what kind of chronic conditions when the video says “chronic conditions” - Importance of having a diverse group. | | | Positive:   - None stated   Areas for Improvement:   - Can be too wordy | TaVNS vs. TMS:   - TaVNS because it was stated it was for more chronic pain disorders. |  |  |
|  | *Key Themes Across Participants* | | - TaVNS was the preference of treatment options. - There was less clarity on TMS and the treatment of pain compared to TaVNS. | | | | | | | | | | | | | | |  |  |
| Spanish  Round 1 Interviews: Video Feedback | | Participant | | | What Was Learned from the Video | | | | Positive Video Feedback | | | | | | Negative Video Feedback or Additional Information | | | | |
|  |  | CAB1 | | | - Learned about the vagus nerve and that she feels it could be promising alternative to medication. | | | | - Enjoyed the visual of the body and the vagus nerve information. - Information on the side effects was enough, but also did not make her think it could be dangerous. | | | | | | - Unable to concentrate after 30 seconds– make it shorter to translate to the Hispanic community. - Subtitles could be helpful to keep focus. - Narration was slow, tropical music. - Make the setting in a town/clinic – Many Hispanics think “UM is for rich people” | | | | |
|  |  | CAB2 | | | - Learned the most from the vagus nerve and body image. | | | | - Informational and clear - Actors seemed like real people, and they did not seem “too perfect” like other medical ads. | | | | | | - More encouraging/inviting. - “Come see for yourselves, this study could work for you.” | | | | |
|  |  | P-S1 | | | - That it is not approved by the FDA - Small risk of skin irritation | | | | - Clear - Feel it could bring something positive to future patients. | | | | | | - NA | | | | |
|  |  | P-S2 | | | - Learned that it can have wide effects. - Learned most from body diagram. | | | | - Video was clear. | | | | | | - Would like to know how long the study takes and if it produces long term relief. - Felt curious, but also uncertainty because of origin/immigration status. | | | | |
|  |  | P-S3 | | | - Learned that the treatment transmots electricity to the nerve that runs through the entire body. - Learned the most from the vagus nerve diagram. | | | | - Flowed well, clear - Felt natural | | | | | | - Minimal feedback because the video was clear. | | | | |
|  |  | *Key Themes Across Participants* | | | - *Focus on the "how it works" and animation.* - *Video and content were clear* | | | | | | | | | | | | | | |
|  | | | | | *Decision Log for Video Enhancement (Based on Feedback):* | | | | | | | | | | | | | | |
|  |  |  |  |  | No Action | | | | | | | - Make it shorter - Narration was slow - Make the setting in a town/clinic | | | | | | | |
|  | | | | | Action | | | | | | | - Concern about not being FDA approved (uncertainty) | | | | | | | |

| Spanish  Round 2 Interviews: Feedback and Expectations |  | | TaVNS | | | | | TMS | | | | | Preference | |
| --- | --- | --- | --- | --- | --- | --- | --- | --- | --- | --- | --- | --- | --- | --- |
|  | Participant | | What Was Learned from the Video | | Video Feedback | Brochure Feedback | | What Was Learned from the Video | | | Video Feedback | Brochure Feedback |  | |
|  | P-S3 | | - Did not state. | | Positive:   - Non-invasive - Safe   Areas for Improvement:   - Add number of sessions someone might need. - Q&A sessions. | Positive:   - Good flow.   Areas for Improvement:   - Q/A section at the end. | | - Learned about brain function balance. - TMS is already used for depression. - Not FDA approved. - <2% have seizures. | | | Positive:   - Clear.   Areas for Improvement:   - More detail on mechanism. - Language might not be clear on how electrical current translates to pain reduction. - More color, more pictures of the treatment. | Positive:   - Not specifically stated.   Areas for Improvement:   - Clarify FDA approval of invasive vagus nerve stimulation. - Add Q&A section. - Include diagram. | TaVNS vs. TMS:   - TaVNS because better explanation for pain. - Would do TaVNS at home but prefers facilities.   Video vs. Brochure:   - Video | |
|  | P-S4 | | - Minimal risk - Study is helping people with chronic pain by stimulating vague nerve. - Decreases pain by stimulating other parts, gets you out of stress mode from pain and inflammation. | | Positive:   - Pill could have more side effects than this treatment, so the treatment is safe. - Rather try this than taking pills.   Areas for Improvement:   - None. | Positive:   - Clear - Straightforward.   Areas for Improvement:   - Catchier title. - Increased clarity why someone needs this. - Order of effects: pain first. - In Spanish: “safety” is not a good translation. | | - It helps mental disorders, depression, anxiety, smoking. - It helps parts of your brain that weren’t working or functioning like they used to be. | | | Positive:   - None stated.   Areas for Improvement:   - Needs descriptions of the parts of the brain and why. - More specific to pain. - Seizure needs to be mentioned more discreetly to avoid fear. | Positive:   - None stated.   Areas for Improvement:   - Title - The word “convulsion” - Picture with the cap on was not in video and not talked about. | TaVNS vs. TMS:   - TaVNS in the home.   Video vs. Brochure:   - Video | |
|  | P-S5 | | - How the neuro-system can stimulate pain in the hips. - Can be a combination of emotional and pain state. | | Positive:   - Interesting. - Very clear. - Side effects clear and not drastic.   Areas for Improvement:   - Time frame that the study has been going on. - Adding assurance: “You could feel this and that” | Positive:   - Good - Self-explanatory.   Areas for Improvement:   - Wording of “physical injuries” under e-stim, confused because their injury is chronic without an injury. | | - It’s been going on for 15 years. - Treats depression, compulsive disorder, smoking. - Connection between brain and hands. | | | Positive:   - Clear. - Balancing of brain caught attention.   Areas for Improvement:   - Mention how much it has been looked at in chronic pain. | Positive:   - Good   Areas for Improvement:   - How long TMS takes. | TaVNS vs. TMS:   - TaVNS because it seems to address pain and less the emotional components.   Video vs. Brochure:   - Video | |
|  | *Key Themes Across Participants* | | - TaVNS was the preference of treatment options. - All of the participants preferred learning from the video over the brochure. - Wording and specific word choice were important in both the video and brochure. - TaVNS was discussed consistently with the potential for pain management, while participants either wanted a better understanding of TMS and pain or only discussed connections between TMS and depression, anxiety, and smoking. | | | | | | | | | | | |
| Haitian-Creole  Round 1 CAB and Interviews: Video Feedback | | Participant | | What Was Learned from the Video | | | Positive Video Feedback | | | Negative Video Feedback or Additional Information | | | |  |
|  |  | CAB1-C1 | | - Learned the process of the study. - Not approved by FDA - What effect it has on you and on the pain. - Works for the brain, so does it “work on your brain to make you try to forget the pain like many pills.” | | | - No specific positive feedback | | | - Need to include more Haitians and black people to get the attention of the Haitian audience. - Series of technical terms that might be difficult. - Participation would depend on the results of tests that have already been done. - Participation would depend on how long the research lasts. - The community would be interested if it was accessible to them because “Most people you meet are always in pain.” | | | |  |
|  |  | CAB1-C2 | | - All the signals are from your brain and shared to all parts of the body. - That research is already being done to see how VaVNS works. | | | - No specific positive feedback | | | - The video narration is too generic (like Google translate). - Would like to know more results of the tests, and how the research was done, to participate. - Compensation would be important in community participation. | | | |  |
|  |  | P-C1 | | - Vagus nerve is where sensation comes from - Pain is felt through the nerve - TaVNS is there to do research | | | - Clear and to the point. | | | - Using other platforms for more awareness - Would be interested in participating to help others from the community. | | | |  |
|  |  | P-C2 | | - There are many people that have pain (high demand) based on the need for this research - The University has something that can change your thinking and pain - Pain can be physical or mental | | | - This video would be helpful in having more individuals from the Haitian community participate | | | - More Information on how the machine works - Interview the person in the video - Make sure the language is more Creole and not French.   “the person who took the treatment to talk more about what he felt.” | | | |  |
|  |  | CAB1-C3 | | - Vagus nerve is the longest nerve in the body - The electrical shocks will be able to help with the pain | | | - The images were helpful in the descriptions - Broke down the abbreviations well so that they were understandable | | | - No specific negative feedback | | | |  |
|  |  | *Key Themes Across Participants* | | - *Video would be helpful in participation from the Haitian community.* - *Clear and images were helpful* - *Language is important: not too much French or Google translate.* | | | | | | | | | |  |
|  | | | | *Decision Log for Video Enhancement (Based on Feedback):* | | | | | | | | | |  |
|  |  |  |  | No Action | | | | |  | | | | |  |
|  | | | | Action | | | | |  | | | | |  |

| Haitian-Creole  Round 2 Interviews: Feedback and Expectations |  | TaVNS | | | TMS | | | Preference |
| --- | --- | --- | --- | --- | --- | --- | --- | --- |
|  | Participant | What Was Learned from the Video | Video Feedback | Brochure Feedback | What Was Learned from the Video | Video Feedback | Brochure Feedback |  |
|  | P-C3 | - TaVNS can be used for pain instead of medication - How TaVNS relates to other chronic medical issues | Positive:   - This could be in place of medication. - Something new to help with chronic disease.   Areas for Improvement:   - No specific areas of improvement - Questions about if it works for many chronic disease and is it readily available. | Positive:   - Good; can use it to ask your doctor more information.   Areas for Improvement:   - No specific areas of improvement - Questions about frequency of use. - “if it is attached to the ear, how is it going to work to affect the body?” | - TMS is for people with anxiety, mental problems, depression. | Positive:   - None stated.   Areas for Improvement:   - No specific areas of improvement. - Has more specific questions after the video about what it is used for and how. | Positive:   - Prefers the brochure because likes to read and understand.   Areas for Improvement:   - None stated, but learned that TMS is for smoking, seizures, depression and anxiety. | TaVNS vs. TMS:   - TaVNS because TMS is for anxiety, depression, and seizure. - TaVNS in the home.   Video vs. Brochure:   - Brochure |
|  | P-C4 | - What part of your body is connected (brain, ear) and how it will help with pain and mental issues. | Positive:   - Video is clear - Clear process of the study   Areas for Improvement:   - More time to read the Creole and listen to the video | Positive:   - Good explanations   Areas for Improvement:   - None stated | - TMS sends signals from the brain to the fingers. - Did not know about TMS previously, learned it can treat depression, smoking, fatigue. | Positive:   - Has been proven safe since it has been around 15 years.   Areas for Improvement:   - None stated - Advice on how to engage those in the community to study and understand the device. | Positive:   - Clear, well-explained.   Areas for Improvement:   - None stated | TaVNS vs. TMS:   - TaVNS in the home. - Ultimately prefers what him and his doctors decide he is the best candidate for.   Video vs. Brochure:   - Brochure |
|  | P-C5 | - All segments were useful in learning. - TaVNS can help manage pain. | Positive:   - Video is clear and concise - Because she can explain the process based on the video, is not scared of the treatment.   Areas for Improvement:   - None stated. | Positive:   - Covers everything   Areas for Improvement:   - None stated | - TMS stimulates the brain to help you feel better. - Learned the most when the video showed the picture of the brain and the instrument. | Positive:   - Clear - Makes people comfortable - Good to help depression and smoking   Areas for Improvement:   - None stated | Positive:   - Flow like the video.   Areas for Improvement:   - None stated | TaVNS vs. TMS:   - TaVNS in the clinic.   Video vs. Brochure:   - Video |
|  | *Key Themes Across Participants* | - TaVNS was the preference of treatment options. - 2 of the 3 participants preferred brochure over the video to research more and be able to discuss with providers. - TaVNS was consistently discussed with the potential for pain treatment while TMS was discussed in connection with other medical conditions or behaviors (i.e. depression, anxiety, smoking.) | | | | | | |
