# Supplementary material for: Using Participatory Methods to Create Informational Videos for Inclusive Brain Stimulation Research Recruitment: Action Research Study and Pilot Randomized Controlled Trial
Source: J Particip Med. 2026 Feb 9;18:e79311. doi: 10.2196/79311 (PMC12885457; doi:10.2196/79311)
Supplement: Multimedia Appendix 2 [file jopm-v18-e79311-s002.docx]

Table 3. Video links

| **taVNS Videos** | | |
| --- | --- | --- |
| **English** | https://www.youtube.com/playlist?list=PL8ykvJ0773K3zCEyNu9a8F_Cf0VxD-u9P | 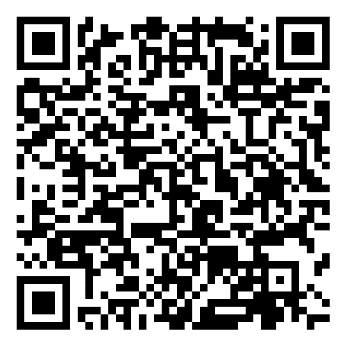 |
| **Spanish** | https://www.youtube.com/playlist?list=PL8ykvJ0773K3ElRkRBqnP2w44CatPqMks | 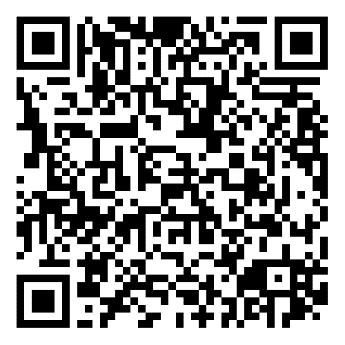 |
| **Haitian-Creole** | https://www.youtube.com/playlist?list=PL8ykvJ0773K0ncZpjLg5Zo3oefh_apkj_ | 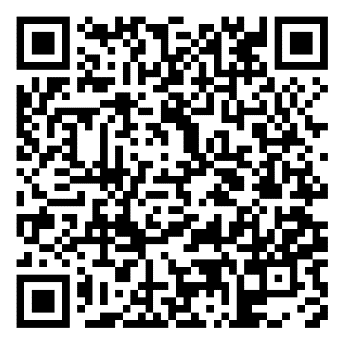 |
| **TMS Videos** | | |
| **English** | https://www.youtube.com/playlist?list=PL8ykvJ0773K2cZs34BHgn1aWLdaTS64Ci | 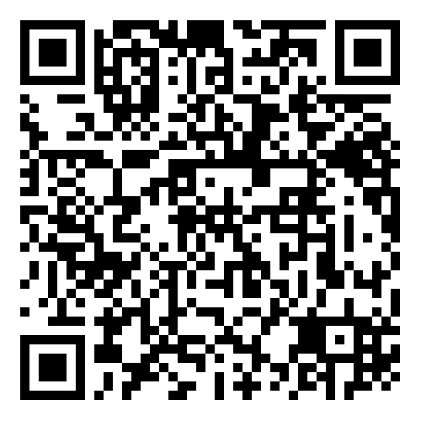 |
| **Spanish** | https://www.youtube.com/playlist?list=PL8ykvJ0773K031N63Kz8uNjPBTH0bY9Hs | 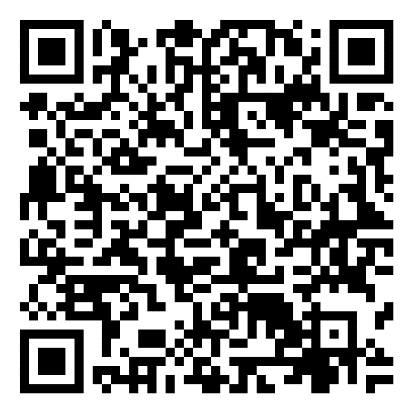 |
